# Nonlinear Relationship Between Vital Signs and Hospitalization/Emergency Department Visits Among Older Home Healthcare Patients and Critical Vital Sign Cutoff for Adverse Outcomes: Application of Generalized Additive Model

**DOI:** 10.1177/10547738251336488

**Published:** 2025-05-13

**Authors:** Se Hee Min, Jiyoun Song, Lauren Evans, Kathryn H. Bowles, Margaret V. McDonald, Sena Chae, Sridevi Sridharan, Yolanda Barrón, Maxim Topaz

**Affiliations:** 1 University of Pennsylvania School of Nursing, Philadelphia, USA; 2 Visiting Nurse Service of New York, USA; 3 University of Iowa College of Nursing, USA; 4 Columbia University School of Nursing, USA

**Keywords:** Adverse outcome, aging, biomarker, home healthcare, older adults

## Abstract

Previous studies have focused on identifying risk factors for older adults receiving home healthcare services without considering vital signs. This may provide important information on deteriorating health conditions that may lead to hospitalization and/or emergency department (ED) visits. Thus, it is important to understand the relationship between vital signs and hospitalization and/or ED visits and critical vital sign points for mitigating the higher risks of hospitalization and/or ED visits. This secondary data analysis uses cross-sectional data from a large, urban home healthcare organization (*n* = 61,615). A generalized additive model was used to understand the nonlinear relationship between each vital sign and hospitalization and/or ED visits through three unadjusted and adjusted models, and to identify a critical vital sign point related to a higher risk of hospitalization and/or ED visits. A significant nonlinear relationship (effective degree of freedom >2.0) was found between systolic, diastolic blood pressure, heart rate, hospitalization, and/or ED visits. The critical inflection point for systolic blood pressure was 120.36 (SE 3.625, *p* < .001), diastolic blood pressure was 72.00 (SE 3.108, *p* < .001), and heart rate was 83.24 (SE 1.994, *p* = .052). Among all vital signs, the risk of hospitalization and/or ED visits sharply increased when an older adult’s heart rate surpassed 83.24 bpm. Our findings reveal that vital signs may serve as a critical indicator of a patient’s clinical condition, especially related to hospitalization and/or ED visit. Clinicians need to be cognizant of these critical thresholds for each vital sign and monitor any deviations from baseline to preempt adverse outcomes.

## Introduction

With the aging population, the number of older adults is increasing rapidly and is expected to double between 2020 and 2050 (Naja et al., 2017). This demographic shift is concomitant with an escalating demand for home healthcare services, particularly to manage the needs of older adults with chronic illnesses (Goodridge et al., 2012; Landers et al., 2016). Currently, an estimated 5 million adults are receiving home healthcare services, which is projected to increase due to a higher prevalence of medical comorbidities among older adults (Landers et al., 2016). Home healthcare services include intermittent home visits by licensed healthcare providers (e.g., nurses, physical/occupational therapists, social workers) to treat and manage medical conditions and promote wellness in their homes (Tao et al., 2012). Much effort has been focused on preventing health deterioration and reducing the risk of adverse events such as hospitalization and emergency department (ED) visits for those receiving home healthcare services (CMS, 2019; Solberg et al., 2018). However, 17.6% of patients receiving home healthcare services are hospitalized or visit an ED during a typical 60-day episode of care (Research Institute for Home Care, 2023). It is estimated that up to 30% of hospitalizations or ED visits among this population could be averted through timely interventions (O’Connor et al., 2014; Solberg et al., 2018). Therefore, there is imperative to deepen our understanding of strategies that can effectively prevent these adverse outcomes.

Previous studies have often focused on identifying risk factors for hospitalization and ED visits among home healthcare patients (Hobensack et al., 2023; Song et al., 2022). For example, Song et al. (2022) identified clusters of risk factors in home healthcare and examined the relationship between these clusters and hospitalization and ED visits (Song et al., 2022). Three distinct risk factor clusters were identified in the study: Cluster 1, characterized by “impaired physical comfort with pain”; Cluster 2, marked by a “high comorbidity burden”; and Cluster 3, which exhibited “impaired cognitive/psychological and skin integrity.” Of these, Cluster 3 had the highest likelihood of hospitalization or ED visits within a 60-day episode of care. In a parallel study, Hobensack et al. (2023) employed advanced data analytics to discern social risk factors, such as education, literacy, physical environment, food insecurity, social environment, and access to care. Each of these factors was found to have a significant association with hospitalization and ED visits. While these studies offer valuable insights into identifying risk factors, they did not consider the potential utility of objective measures, such as vital signs (e.g., heart rate, blood pressure, temperature, and respiratory rate), which are important indicators of a patient’s clinical condition (Chester & Rudolph, 2011). These physiological parameters could offer critical information to enhance the understanding of risk factors and guide more targeted interventions (Chester & Rudolph, 2011).

Vital signs collected at home healthcare admission and during each subsequent home visit produce valuable data. As vital signs serve as critical indicators of a patient’s clinical condition, it is important to include them in predictive models and understand their association with the risk of hospitalization and/or ED visits (Amer et al., 2020; Brekke et al., 2019; Chester & Rudolph, 2011). Specifically, these vital signs provide important information on any deteriorating health conditions that might lead to hospitalization and ED visits and allow clinicians to implement timely interventions to reduce adverse health outcomes (Amer et al., 2020; Brekke et al., 2019). However, limited studies have focused on understanding the single cutoffs or normal/abnormal range for vital signs among older adults. As older adults experience multiple age-related changes in their vital signs due to molecular changes, structural and organ system changes, systemic changes, and altered compensation to stressors (Chester & Rudolph, 2011), a nuanced understanding of these age-specific vital sign cutoffs is essential to mitigate the higher risks of hospitalization and ED visits.

Associations between vital signs and adverse outcomes have been traditionally studied with generalized linear models (GLMs) (Zhao, 2013); however, these models have certain limitations. While GLMs can be adapted to examine nonlinear relationships by including quadratic terms, this approach raises significant multicollinearity issues (Tanskanen et al., 2016). Such limitations are particularly relevant when dealing with real-world data that often exhibit complex, nonlinear relationships among variables. To address these challenges, this exploratory study uses generalized additive models (GAMs) to investigate the possible nonlinear relationships between vital signs and the risk of hospitalization or ED visits and the degree of nonlinearity among older home healthcare patients before and after adjusting for sociodemographic, lifestyle, and clinical characteristics (T. J. Hastie, 1992; Horowitz, 2001). The use of GAM will allow us to identify the critical thresholds, or inflection points, for each vital sign that correlates with an elevated risk of these adverse outcomes of hospitalization or ED visits.

## Methods

### Study Sample and Dataset

This is a secondary data analysis of Medicare beneficiaries admitted to home healthcare services at one of the largest home healthcare agencies in the northeastern U.S. between January 1, 2015, and December 31, 2017. Data included the Outcome and Assessment Information Set (OASIS) C1 version, which is a standardized assessment tool that measures quality and outcome for home healthcare patients and includes nearly 100 items related to sociodemographic information, mental and cognitive health, head-to-toe assessment, functional status, and service needs (O’Connor & Davitt, 2012). Home healthcare clinicians are required to collect OASIS data for all Medicare and Medicaid patients during admission, discharge, transfer, any change in condition, and recertifications every 60 days. The current study used the OASIS data collected at the time of admission.

### Measures

#### Sociodemographic, Lifestyle, and Clinical Characteristics

The sociodemographic, lifestyle, and clinical characteristics used in this study were selected from previous studies (Hobensack et al., 2023; Song et al., 2022) and obtained from the OASIS and administrative data at the start of care. We used a total of 27 OASIS variables which include age, race, gender, type of insurance, living situation, current smoking status, current alcohol consumption, current drug activities, presence of diabetes, hypertension, depression, dementia, cancer, heart failure, pulmonary disease, renal failure, skin ulcer, urinary incontinence, obesity, recent falls in the past 12 months, altered mental status in the past 3 months, multiple hospitalizations in the past 6 months, taking more than five medications, overall functional status, length of stay, and the degree of assisted activities of daily living (ADL) needed and severity.

#### Vital Signs

Vital signs (i.e., systolic blood pressure, diastolic blood pressure, heart rate, temperature) at admission (first visit of the episode of care) were obtained from the electronic health records. Home healthcare patients had different numbers of visits and episodes, and had missing vital signs at subsequent visits, which is the main reason we used vital signs at admission collected for all patients. We only selected vital signs that had a nonlinear relationship with our study outcome to conduct GAM based on initial visualization of the relationship and the effective degree of freedom (EDF), which excluded the respiratory rate.

#### Hospitalization and Emergency Department Visits

The main study outcome was hospitalization and/or ED visits that occurred up to 60 days after admission to home healthcare services. This variable was obtained in the follow-up OASIS assessments. The outcome was dichotomized to 0 = no hospitalization and/or ED visit and 1 = at least one hospitalization and/or ED visit.

### Ethical Considerations

All study materials, such as the study dataset, corresponding codebooks, and statistical programs, were stored in an encrypted server at Columbia University and the Visiting Nurse Service of New York. Only the de-identified information was archived and analyzed. The current study was approved by the institutional review boards of Columbia University and the Visiting Nurse Service of New York [IRB# E20-003].

### Data Analysis

We used GAM to estimate the nonlinear relationship between the 60-day hospitalization or ED visits and the vital signs predictors at baseline. GAM is a type of nonparametric regression-based method that allows for modeling the nonlinear relationship between the study outcome and the predictors that do not require pre-specifying the form of the nonlinear relationship (T. J. Hastie, 1992; Horowitz, 2001). The linear relationship between the study outcome and the predictors assumed when using GLM is replaced by several nonlinear smooth functions to capture nonlinear relationships (T. J. Hastie, 1992; Horowitz, 2001). Thus, we applied the nonlinear smooth function to the predictor (e.g., vital signs) and summed the component response of non-parametric means (Wood, 2003). For the nonlinear smooth function, several options exist, such as smoothing spline, polynomial function, step function, and natural cubic splines, which are nonlinear functions that are tied together to construct a GAM model without imposing a penalty for data wiggliness (Wood, 2003). The *mgcv* package v.1.8–31 in the R statistical software program (v. 4.0.5) was used for data analysis. The *mgcv* package uses the thin-plate regression splines as smoothers, which are low-rank isotropic smoothers of any number of covariates as a method to estimate smooth functions within a large, complex dataset and to prevent model overfitting (Wood, 2003). We estimated the smoothing parameter based on a restricted maximum likelihood (REML) approach, which is preferred for finite sample sizes to further prevent model overfitting. The basis dimension used to represent the smoothing term (*k*), which is the maximum possible degrees of freedom allowed for a smooth term in a model, was set to 6 after verifying with the “gam.check” function available in the *mgcv* package, indicating that the selected smoothing term is appropriate to capture the nonlinearity in our data (T. J. Hastie, 1992; Horowitz, 2001). When building three unadjusted and adjusted GAM models, we used family = binomial and link function = logit as the study outcome was binary (T. Hastie & Tibshirani, 1995; Horowitz, 2001; Lee et al., 2023; López-Moreno & Nogués-Bravo, 2005). The unadjusted GAM models examine the nonlinear relationship between each vital sign and hospitalization and ED visits, while the adjusted GAM models control for important covariates and provides additional values for predicting hospitalization and ED visits (Lee et al., 2023; Min et al., 2022). For the three GAM models for each vital sign, Model 1 was not adjusted for any covariates (vital sign only), Model 2 was adjusted for sociodemographic factors, and Model 3 was adjusted for sociodemographic, lifestyle, and clinical factors.

The predicted smooth functions and confidence intervals were plotted for each GAM model. In addition, the EDF value was obtained, reflecting the degree of nonlinearity of the relationship. Typically, an EDF = 1 shows a linear relationship, 1 < EDF ≤ 2 shows a weak nonlinear relationship, and EDF >2 shows a highly nonlinear relationship (Hunsicker et al., 2016). We used the segmented package’s two-piecewise linear regression model to understand the critical inflection point where the slope significantly changed direction when there was a nonlinear relationship based on EDF value (Muggeo, 2008). Identifying a critical inflection point is important because it provides information on where the risk of hospitalization and ED visits increases drastically related to each vital sign.

## Results

### Participant Characteristics

Our study sample included a total of 61,615 older adults with a mean age of 80.06 years (SD 9.43). More than half (64.79%) were White, and the remainder were Black (15.88%), Hispanic (12.78%), and Asian/Pacific Islander (6.55%). There were more females (63.82%) than males (36.18%), with more than half living with another person (58.23%). The highest prevalence of clinical conditions was found in hypertension (65.50%), followed by diabetes (28.19%). Nearly one-quarter of older adults reported recent falls in the past 12 months (21.21%) and multiple hospitalizations in the past 6 months (20.92%). In addition, the majority took more than five medications at admission (78.53%). The overall functional status of our study sample was likely to be stable (78.69%), with the degree of ADL assistance needed ranging from 0 to 9, to be 8.03 (SD 1.52), and the severity ranging from 0 to 38, to be 15.13 (SD 6.44). The higher score for the degree of ADL assistance and severity indicates a higher degree and severity. The mean length of stay was 41.15 days (SD 58.95). The mean diastolic blood pressure at baseline was 70.9 mmHg (SD 8.18), systolic blood pressure 123.7 mmHg (SD 14.08), and heart rate 74.96 bpm (SD 8.94) (Table 1).

**Table 1.** Baseline Characteristics of the Study Sample.

Variables	*n* (%) or mean ± SD		
Diastolic, mmHg	70.9 ± 8.18		
Systolic, mmHg	123.7 ± 14.08		
Heart Rate, bpm	74.96 ± 8.94		
Hospitalization/ED Visit			
Yes	10,690 (17.54)		
Sociodemographic factors		Clinical factors	
Age	80.06 ± 9.43	Diabetes (yes)	17,370 (28.19)
Race		Hypertension (yes)	40,359 (65.50)
White	39,922 (64.79)	Depression (yes)	4,846 (7.86)
Black	9,787 (15.88)	Dementia (yes)	7,699 (12.49)
Hispanic	7,877 (12.78)	Cancer (yes)	1,241 (2.01)
Asian/Pacific Islander	4,029 (6.55)	Heart (yes)	7,838 (12.72)
Gender		Pulmonary (yes)	8,736 (14.17)
Male	22,298 (36.18)	Renal (yes)	2,273 (3.68)
Female	39,317 (63.82)	Skin ulcer (yes)	6,793 (11.02)
Insurance		Genitourinary (yes)	8 (0.0001)
Medicare	61,554 (99.90)	Obesity (yes)	7,640 (12.39)
Living situation		Recent falls (yes)	1,3069 (21.21)
Alone	23,977 (38.91)	Altered mental status (yes)	9,041 (14.67)
With another person	35,882 (58.23)	Multiple hospitalizations (yes)	12,890 (20.92)
In a congregate situation	1,740 (2.86)	Taking more than 5 medications (yes)	48,388 (78.53)
*Lifestyle factors*		Overall status	
Smoking (yes)	4,016 (6.51)	Stable	4,434 (7.19)
Alcohol (yes)	685 (1.11)	Likely to be stable	48,486 (78.69)
Drug (yes)	262 (0.04)	Fragile	8,368 (13.58)
		Serious	327 (0.54)
		ADL needed	8.03 ± 1.52
		ADL severity	15.13 ± 6.44
		Length of stay	41.15 ± 58.95

### Generalized Additive Models (Systolic Blood Pressure)

#### Systolic BP Model 1: Not Adjusting for Covariates

**Figure 1.**
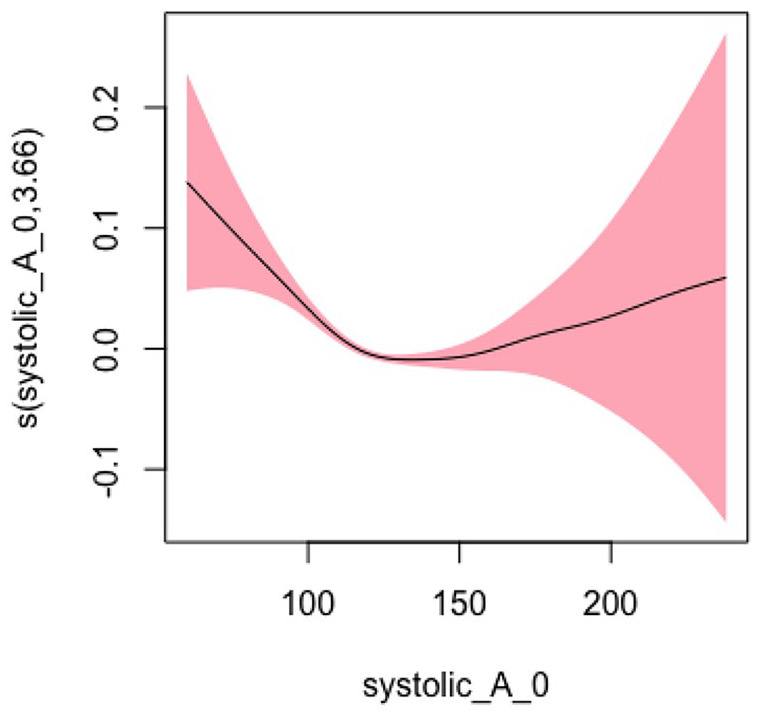
Unadjusted model (systolic). *Note. X*-axis = systolic blood pressure; *Y* **-**axis = risk of hospitalization and/or ED visits.

In Model 1, we did not adjust for any covariates and used the smoothing spline function of systolic blood pressure as a predictor variable and hospitalization and/or ED visits as the study outcome. The EDF was 3.66, which indicated a statistically significant and highly nonlinear relationship as represented by the U-shape of the curve (*p* < .001). Figure 1 shows the plots of estimated smoothing spline functions for Model 1.

#### Systolic BP Model 2: Adjusted for Sociodemographic Characteristics

**Figure 2.**
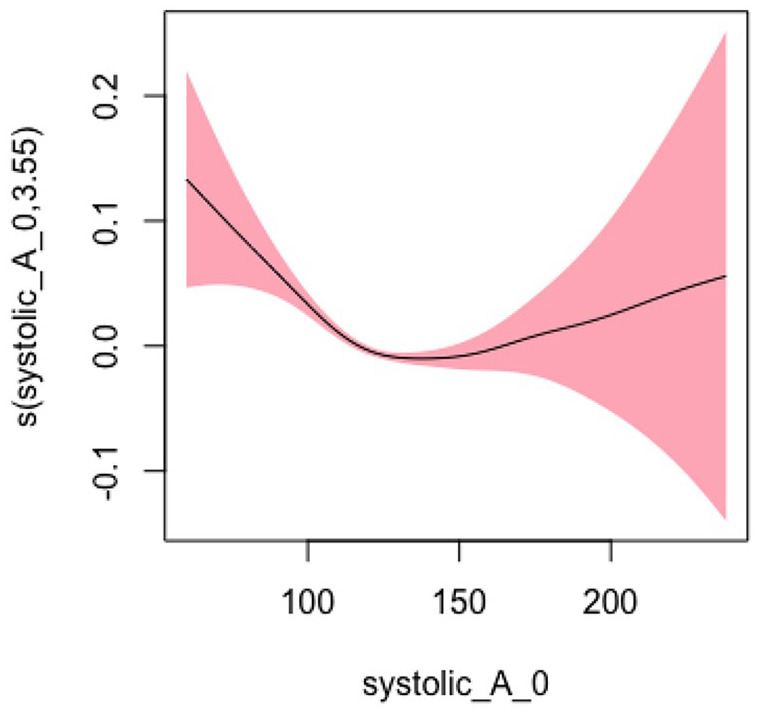
Adjusted model (systolic) for sociodemographic characteristics. *Note. X*-axis = systolic blood pressure; *Y* **-**axis = risk of hospitalization and/or ED Visits.

In Model 2, adjusting for sociodemographic factors, the EDF was 3.55, slightly decreasing from Model 1 but still indicating a statistically significant and highly nonlinear relationship (*p* < .001). Figure 2 shows the plots of estimated smoothing spline functions for Model 2.

#### Systolic BP Model 3: Adjusted for Sociodemographic, Lifestyle, and Clinical Characteristics

**Figure 3.**
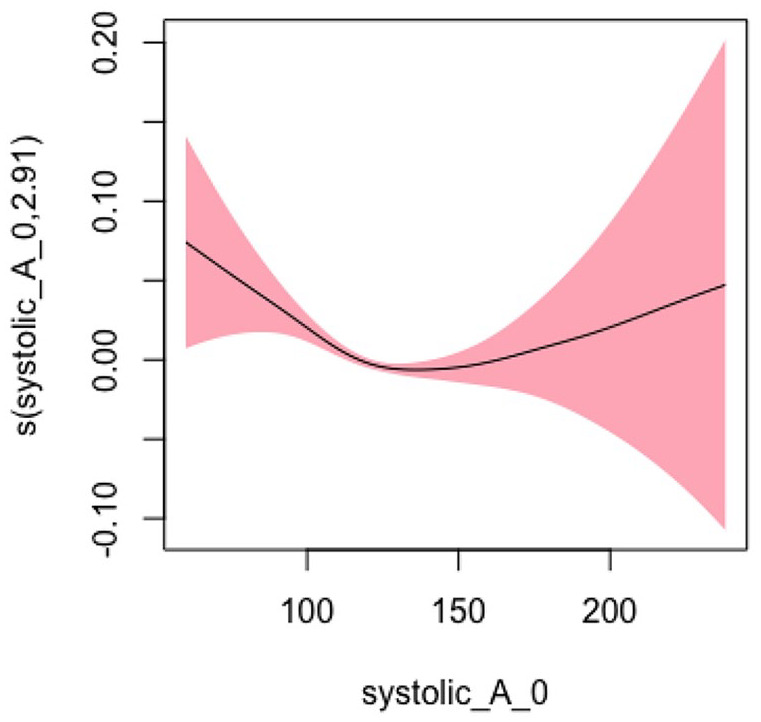
Adjusted model (systolic) for all covariates. *Note. X*-axis = systolic blood pressure; *Y* **-**axis = risk of hospitalization and/or ED Visits.

In Model 3, adjusting for sociodemographic, lifestyle, and clinical ***characteristics*** factors, the EDF was 2.913, which decreased from previous models but still indicated a statistically significant and highly nonlinear relationship (*p* < .001). Figure 3 shows the plots of estimated smoothing spline functions for Model 3 (Table 2).

**Table 2.** Estimated Regression Coefficients from the Overall Additive Model with a Factor-smooth Interaction.

		Model 1		Model 2		Model 3	
		Estimate	*p*-value	Estimate	*p*-value	Estimate	*p*-value
Intercept		0.173	<.001^***^	0.271	.034^*^	0.156	.220
Systolic X Hospitalization/ED Visit(smooth)		3.66	<.001^***^	3.55	<.001^***^	2.913	<.001^***^
Sociodemographic factors							
Age	Continuous			0.001	<.001^***^	0.0001	.788
Race	White			Ref		Ref	
	Black			0.046	<.001^***^	0.021	<.001^***^
	Hispanic			0.034	<.001^***^	0.015	.006^**^
	Asian/Pacific Islander			−0.011	.146	−0.018	.019^*^
Gender	Male			Ref		Ref	
	Female			−0.034	<.001^***^	−0.024	<.001^***^
Insurance (Medicare)	Yes (ref = no)			−0.094	.243	−0.054	.511
Living situation (alone)	Yes (ref = no)			−0.055	.572	−0.046	.626
Living situation (in a congregate situation)	Yes (ref = no)			−0.013	.898	−0.019	.839
Living situation (with others)	Yes (ref = no)			−0.048	.626	−0.053	.573
Lifestyle factors							
Smoking	Yes (ref = no)					0.009	0.237
Alcohol	Yes (ref = no)					0.028	0.122
Drug	Yes (ref = no)					0.036	0.205
Clinical factors							
Diabetes	Yes (ref = no)					0.036	<.001^***^
Hypertension	Yes (ref = no)					−0.013	.001^**^
Depression	Yes (ref = no)					0.002	.819
Dementia	Yes (ref = no)					−0.022	<.001^***^
Cancer	Yes (ref = no)					0.135	<.001^***^
Heart	Yes (ref = no)					0.075	<.001^***^
Pulmonary	Yes (ref = no)					0.026	<.001^***^
Renal	Yes (ref = no)					0.083	<.001^***^
Skin ulcer	Yes (ref = no)					0.129	<.001^***^
Genitourinary	Yes (ref = no)					−0.129	.481
Obesity	Yes (ref = no)					−0.002	.681
Falls	Yes (ref = no)					0.008	.055
Altered mental status	Yes (ref = no)					0.016	.003^**^
Multiple hospitalization	Yes (ref = no)					0.069	<.001^***^
Taking more than 5 medications	Yes (ref = no)					0.016	<.001^***^
Overall status	Stable					Ref	
	Likely to be stable					0.011	.101
	Fragile					0.027	<.001^***^
	Serious					0.033	.202
ADL needed	Continuous					−0.003	.063
ADL severity	Continuous					0.005	<.001^***^
Length of stay	Continuous					0.106	<.001^***^
		R-sq. (adj) = 0.00139, DE = 0.148%, REML = 18,151		R-sq. (adj) = 0.00568, DE = 0.599%, REML = 18,095		R-sq. (adj) = 0.0545, DE = 5.56%, REML = 16,717	

* *p* < .05, ** *p* < .01, *** *p* < .001

*Note.* EDF = effective degree of freedom, DE = deviance explained, REML = restricted maximum likelihood, R-sq = R-squared.

### Generalized Additive Models (Diastolic Blood Pressure)

#### Diastolic BP Model 1: Not Adjusting for Covariates

**Figure 4.**
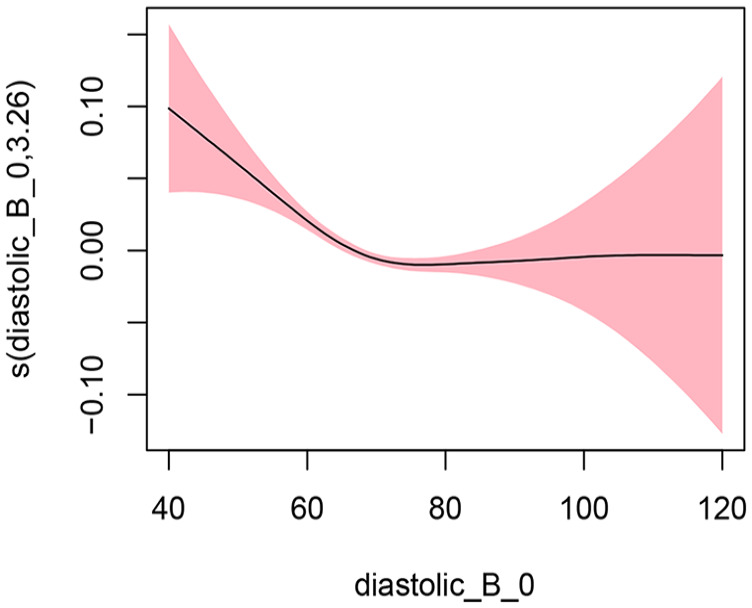
Unadjusted model (diastolic). *Note. X*-axis = diastolic blood pressure; *Y*-axis = risk of hospitalization and/or ED visits.

In Model 1, we did not adjust for any covariates and used the smoothing spline function of systolic blood pressure as a predictor variable and hospitalization and/or ED visits as the study outcome. The EDF was 3.261, which indicated a statistically significant and highly nonlinear relationship as represented by the U-shape of the curve (*p* < .001). Figure 4 shows the plots of estimated smoothing spline functions for Model 1.

#### Diastolic BP Model 2: Adjusted for Sociodemographic Characteristics

**Figure 5.**
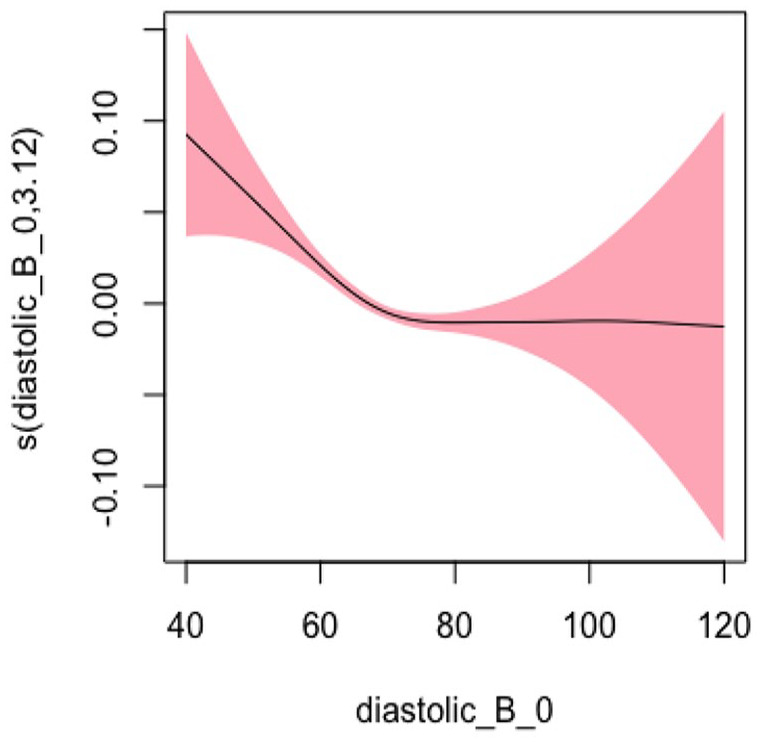
Adjusted model (diastolic) for sociodemographic characteristics. *Note. X*-axis = diastolic blood pressure; *Y*-axis = risk of hospitalization and/or ED visits.

In Model 2, adjusting for sociodemographic factors, the EDF was 3.119, which slightly decreased from Model 1 but still indicated a statistically significant and highly nonlinear relationship (*p* < .001). Figure 5 shows the plots of estimated smoothing spline functions for Model 2.

#### Diastolic BP Model 3: Adjusted for Sociodemographic, Lifestyle, and Clinical Characteristics

**Figure 6.**
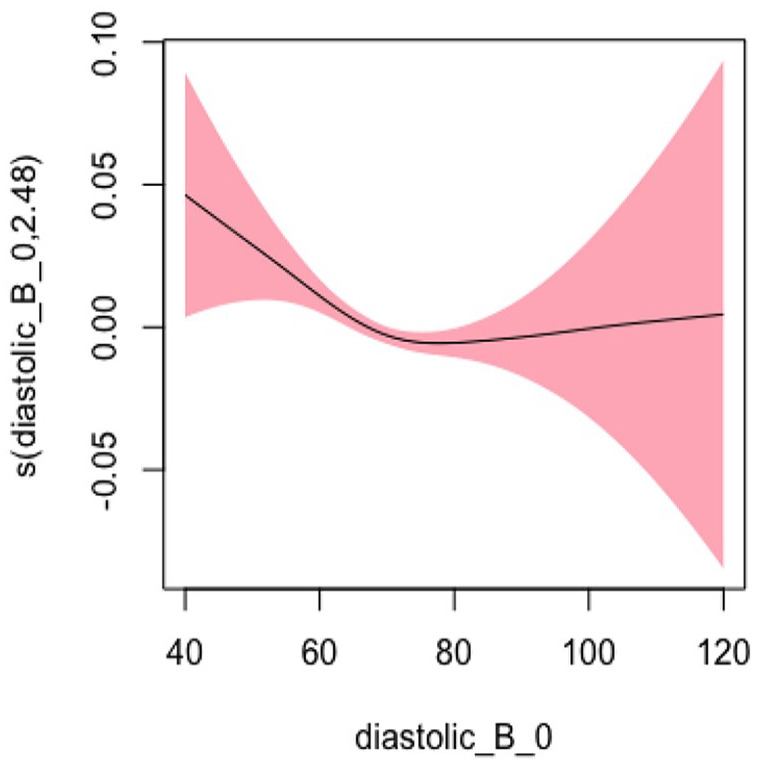
Adjusted model (diastolic) for all covariates. *Note. X*-axis = diastolic blood pressure; *Y*-axis = risk of hospitalization and/or ED visits.

In Model 3, adjusting for sociodemographic, lifestyle, and clinical factors, the EDF was 2.478, which decreased from previous models but still indicated a statistically significant and highly nonlinear relationship (*p* = .01). Figure 6 shows the plots of estimated smoothing spline functions for Model 3 (Table 3).

**Table 3.** Estimated Regression Coefficients from the Overall Additive Model with a Factor-smooth Interaction.

		Model 1		Model 2		Model 3	
		Estimate	*p*-value	Estimate	*p*-value	Estimate	*p*-value
Intercept		0.173	<.001^***^	0.276	.031^*^	0.129	0.302
Diastolic × hospitalization/ED visit (smooth)		3.261	<.001^***^	3.119	<.001^***^	2.478	0.001^**^
Sociodemographic factors							
Age	Continuous			0.0001	^.002**^	0.0001	0.983
Race	White			Ref		Ref	
	Black			0.046	<.001^***^	0.023	<0.001^***^
	Hispanic			0.033	<0.001^***^	0.015	0.006^**^
	Asian/Pacific Islander			−0.012	0.115	−0.017	0.023^*^
Gender	Male			Ref		Ref	
	Female			−0.034	<0.001^***^	−0.024	<0.001^***^
Insurance (Medicare)	Yes (ref = no)			−0.096	0.234	−0.028	0.718
Living situation (alone)	Yes (ref = no)			−0.051	0.601	−0.045	0.637
Living situation (in a congregate situation)	Yes (ref = no)			−0.009	0.926	−0.018	0.852
Living situation (with others)	Yes (ref = no)			−0.043	0.656	−0.052	0.585
Lifestyle factors							
Smoking	Yes (ref = no)					0.011	0.173
Alcohol	Yes (ref = no)					0.024	0.174
Drug	Yes (ref = no)					0.037	0.187
Clinical factors							
Diabetes	Yes (ref = no)					0.036	<0.001^***^
Hypertension	Yes (ref = no)					−0.013	0.001^**^
Depression	Yes (ref = no)					0.001	0.861
Dementia	Yes (ref = no)					−0.022	<0.001^***^
Cancer	Yes (ref = no)					0.135	<0.001^***^
Heart	Yes (ref = no)					0.077	<0.001^***^
Pulmonary	Yes (ref = no)					0.026	<0.001^***^
Renal	Yes (ref = no)					0.084	<0.001^***^
Skin ulcer	Yes (ref = no)					0.128	<0.001^***^
Genitourinary	Yes (ref = no)					−0.127	0.490
Obesity	Yes (ref = no)					−0.003	0.562
Falls	Yes (ref = no)					0.009	0.035^*^
Altered mental status	Yes (ref = no)					0.016	0.003^**^
Multiple hospitalizations	Yes (ref = no)					0.071	<0.001^***^
Taking more than 5 medications	Yes (ref = no)					0.018	<0.001^***^
Overall status	Stable					Ref	
	Likely to be stable					0.011	0.102
	Fragile					0.027	0.001^**^
	Serious					0.024	0.328
ADL needed	Continuous					−0.003	0.047^*^
ADL severity	Continuous					0.005	<0.001^***^
Length of stay	Continuous					0.163	<0.001^***^
		R-sq. (adj) = 0.00128, DE = 0.136%, REML = 18,152		R-sq. (adj) = 0.00552, DE = 0.581%, REML = 18,097		R-sq. (adj) = 0.0546, DE = 5.54%, REML = 17,154	

* *p* < .05, ** *p* < .01, *** *p* < .001

*Note.* EDF = effective degree of freedom, DE = deviance explained, REML = restricted maximum likelihood, R-sq = R-squared.

### Generalized Additive Models (Heart Rate)

#### Heart Rate Model 1: Not Adjusting for Covariates

**Figure 7.**
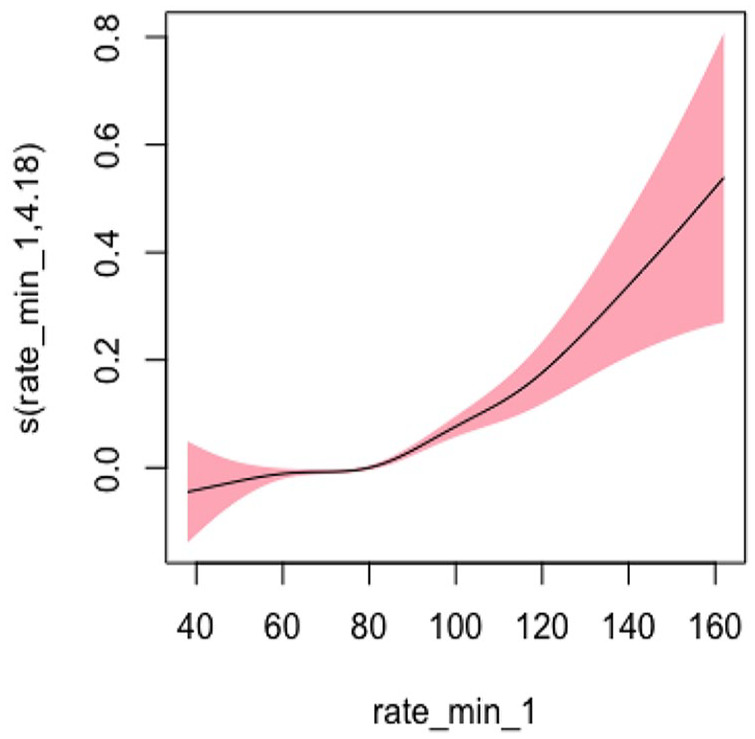
Unadjusted model (heart rate). *Note. X-*axis = heart rate; *Y*-axis = risk of hospitalization and/or ED visits.

In Model 1, we did not adjust for any covariates and used the smoothing spline function of systolic blood pressure as a predictor variable and hospitalization and/or ED visits as the study outcome. The EDF was 4.183, indicating a highly nonlinear relationship that was statistically significant as represented by the U-shape of the curve (*p* < .001). Figure 7 shows the plots of estimated smoothing spline functions for Model 1.

#### Heart Rate Model 2: Adjusted for Sociodemographic Characteristics

**Figure 8.**
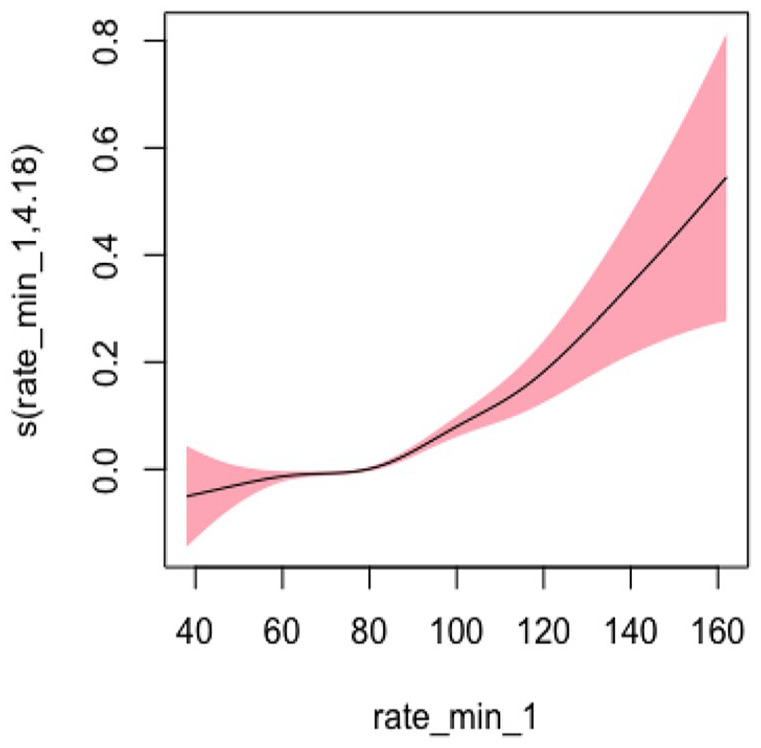
Adjusted model (heart rate) for sociodemographic characteristics. *Note. X-*axis = heart rate; *Y*-axis = risk of hospitalization and/or ED visits.

In Model 2, adjusting for sociodemographic factors, the EDF was 4.184, which is similar to Model 1 and indicated a statistically significant and highly nonlinear relationship (*p* < .001). Figure 8 shows the plots of estimated smoothing spline functions for Model 2.

#### Heart Rate Model 3: Adjusted for Sociodemographic, Lifestyle, and Clinical Characteristics

**Figure 9.**
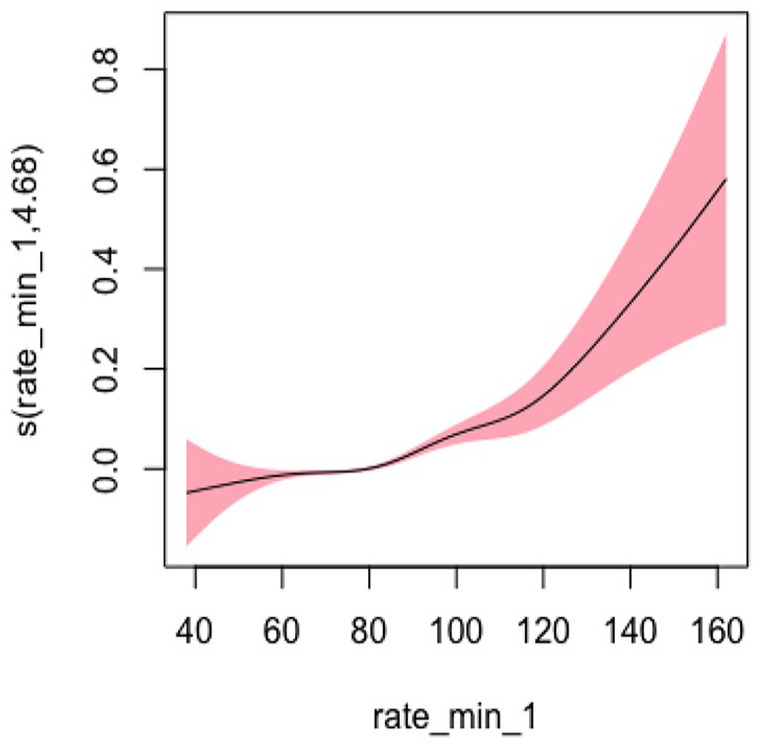
Adjusted model (heart rate) for all covariates. *Note. X-*axis = heart rate; *Y*-axis = risk of hospitalization and/or ED Visits.

In Model 3, adjusting for sociodemographic, lifestyle, and clinical factors, the EDF was 4.676, which was the highest among all three models and indicated a statistically significant and highly nonlinear relationship (*p* < .001). Overall, the degree of nonlinearity for heart rate was the most pronounced compared to other vital signs. Figure 9 shows the plots of estimated smoothing spline functions for Model 3 (Tables 4 and 5).

**Table 4.** Estimated Regression Coefficients from the Overall Additive Model with a Factor-smooth Interaction.

		Model 1		Model 2		Model 3	
		Estimate	*p*-value	Estimate	*p*-value	Estimate	*p*-value
Intercept		0.174	<.001^***^	0.242	.058	0.106	.396
Heart rate × hospitalization/ED visit (smooth)		4.183	<.001^***^	4.184	<.001^***^	4.676	<.001^***^
Sociodemographic factors							
Age	Continuous			0.001	<0.001^***^	0.0002	.296
Race	White			Ref		Ref	
	Black			0.044	<0.001^***^	0.021	<.001^***^
	Hispanic			0.033	<0.001^***^	0.014	.009^**^
	Asian/Pacific Islander			−0.011	0.157	−0.017	.025^*^
Gender	Male			Ref		Ref	
	Female			−0.036	<0.001^***^	−0.025	<.001^***^
Insurance (Medicare)	Yes (ref = no)			−0.092	0.256	−0.027	.735
Living situation (alone)	Yes (ref = no)			−0.049	0.616	−0.042	.659
Living situation (in a congregate situation)	Yes (ref = no)			−0.003	0.968	−0.013	.895
Living situation (with others)	Yes (ref = no)			−0.041	0.677	−0.048	.610
Lifestyle factors							
Smoking	Yes (ref = no)					0.008	.275
Alcohol	Yes (ref = no)					0.021	.243
Drug	Yes (ref = no)					0.037	.182
Clinical factors							
Diabetes	Yes (ref = no)					0.036	<.001^***^
Hypertension	Yes (ref = no)					−0.013	.001^**^
Depression	Yes (ref = no)					0.001	.858
Dementia	Yes (ref = no)					−0.021	<.001^***^
Cancer	Yes (ref = no)					0.128	<.001^***^
Heart	Yes (ref = no)					0.078	<.001^***^
Pulmonary	Yes (ref = no)					0.023	<.001^***^
Renal	Yes (ref = no)					0.085	<.001^***^
Skin ulcer	Yes (ref = no)					0.129	<.001^***^
Genitourinary	Yes (ref = no)					−0.126	.494
Obesity	Yes (ref = no)					−0.003	.558
Falls	Yes (ref = no)					0.009	.039^*^
Altered mental status	Yes (ref = no)					0.016	.003^**^
Multiple hospitalizations	Yes (ref = no)					0.071	<.001^***^
Taking more than 5 medications	Yes (ref = no)					0.018	<.001^***^
Overall status	Stable					Ref	
	Likely to be stable					0.011	.107
	Fragile					0.026	.001^**^
	Serious					0.026	.297
ADL needed	Continuous					−0.002	.067
ADL severity	Continuous					0.005	<.001^***^
Length of stay	Continuous					0.164	<.001^***^
		R-sq. (adj) = 0.0025, DE = 0.266%, REML = 18,151		R-sq. (adj) = 0.00704, DE = 0.736%, REML = 18,091		R-sq. (adj) = 0.0563, DE = 5.72%, REML = 17,143	

* *p* < .05, ** *p* < .01, *** *p* < .001

*Note.* EDF = effective degree of freedom, DE = deviance explained, REML = restricted maximum likelihood, R-sq = R-squared.

**Table 5.** EDF of All Models.

	Model 1. Not adjusting for covariates	Model 2. Adjusted for sociodemographic factors	Model 3. Adjusted for sociodemographic, lifestyle, and clinical factors
Systolic BP Model	3.66***	3.261***	4.183***
Diastolic BP Model	3.55***	3.119***	4.184***
Heart rate model	2.913***	2.478**	4.676***

*** *p* < .001. ** *p* < .01. *p < .05.

*Note.* EDF = effective degree of freedom; an EDF = 1 shows a linear relationship, 1 < EDF ≤ 2 shows a weak nonlinear relationship, and EDF > 2 shows a highly nonlinear relationship.

### Critical Inflection Point

The critical inflection point for systolic blood pressure was 120.36 (SE 3.625) and diastolic blood pressure was 72.00 (SE 3.108), both of which were statistically significant (*p* < .001). In addition, the critical inflection point was 83.24 (SE 1.994) for heart rate (*p* = .052) (Table 6).

**Table 6.** Critical Inflection Point.

	Estimate	Standard error	*p*-value
Systolic	120.36	3.625	<0.001^***^
Diastolic	72.00	3.108	<0.001^***^
Heart rate	83.24	1.994	0.052

Significant covariates included: ***p < .001. **p < .01. *p < .05.

## Discussion

To the best of our knowledge, this is one of the first studies to examine the nonlinear relationship between vital signs at the time of admission and hospitalization and/or ED visits among older adults receiving home healthcare services. The GAM was used to understand the underlying hidden nonlinear relationship between each vital sign and hospitalization and/or ED visit. In addition, this study identified a critical inflection point for each vital sign where the risk of hospitalization and/or ED visits increases significantly.

A significant nonlinear relationship was found between systolic, diastolic, heart rate, hospitalization, and/or ED visits in older home healthcare patients. To date, very limited studies have examined the nonlinear association between vital signs and hospitalization and/or ED visits in the home healthcare setting. However, previous studies have identified a nonlinear relationship between vital signs and adverse health outcomes such as heart failure and mortality in inpatient clinical settings (Anderson et al., 2016; Sun et al., 2021). For example, one of the studies examined the association between the number of abnormal vital signs and in-hospital mortality for patients suffering in-hospital cardiac arrest and presumed a nonlinear relationship when conducting a multivariable logistic regression model (Anderson et al., 2016). The authors found that in-hospital mortality increases with the increasing number of pre-arrest abnormal vital signs and the severity of vital sign derangements (Anderson et al., 2016). While the use of a polynomial function and adding higher-order terms in a multivariable logistic regression model may capture the nonlinear relationship, this may lead to model overfitting where the regression coefficients represent the noise rather than the true relationship between vital signs and the outcome (Ying, 2019). Thus, our study suggests the use of GAM, which offers several advantages (T. J. Hastie, 1992; Horowitz, 2001). First, GAM imposes fewer assumptions about the form of the relationship, thus providing greater interpretability and flexibility (T. J. Hastie, 1992; Horowitz, 2001). Second, GAM includes regularization techniques that mitigate the risk of overfitting, enhancing the model’s predictive validity. Therefore, employing GAM will not only assist in uncovering any potential nonlinear relationships between vital signs and adverse healthcare outcomes but also facilitate the identification of critical inflection points in each vital sign where the risk of such outcomes increases significantly. Overall, our findings and previous studies highlight the potential nonlinearity between vital signs and adverse outcomes such as hospitalization and/or ED visits in home healthcare.

Our study found a critical point for each vital sign where the risk of hospitalization and/or ED visits increases significantly among older home healthcare patients, as represented by the U-shape of the curve. The critical point was 120.36 for systolic blood pressure, 72 for diastolic blood pressure, and 83.24 for heart rate. The existing literature has established that clinicians often attribute abnormal or high vital sign readings in older adults to the well-documented age-related physiological changes (Teixeira et al., 2015). With aging, homeostatic mechanisms change, which may lead to challenges in maintaining internal physiological consistency (Su et al., 2022). For example, older adults experience age-related dysregulated organ function and thus may be unable to respond appropriately to stressors (Su et al., 2022). The prevalence of hypertension increases with age, but the risk of hypotension is high in older adults due to the impaired ability of the cardiovascular system to respond rapidly to stressors and the advancing arterial wall stiffness. While diastolic blood pressure is not significantly affected by aging, the gap between systolic and diastolic blood pressure becomes an issue for older adults (Chester & Rudolph, 2011). In addition, the resting heart rate also increases with age due to deconditioning and autonomic dysregulation (Chester & Rudolph, 2011). As a result, clinicians often assume abnormal or high vital signs to be normal among older adults due to their age-related physiological changes (Teixeira et al., 2015). However, our study findings challenge this conventional assumption. We found that the identified critical point for each vital sign related to the risk of hospitalization and/or ED visits from the GAM models does not significantly differ from younger adults (Teixeira et al., 2015). This may indicate that the factors influencing vital signs may not be exclusively age-specific. Rather, other clinical factors, such as infections, dehydration, or environmental stressors, may affect vital signs in a similar manner across age groups (Teixeira et al., 2015). Thus, clinicians need to refrain from automatically attributing abnormal vital signs in older adults to age-related physiological changes alone and to maintain a vigilant, individualized approach to monitoring and interpreting vital sign parameters in this vulnerable population.

While the EDF was greater than 2 for all vital signs, which indicates high nonlinearity, it is also important to consider the U-shape of nonlinearity. For both systolic and diastolic blood pressure, the nonlinearity is more pronounced below the identified critical point, and the slope flattens beyond the critical point. By contrast, the slope increases drastically beyond the identified critical point for heart rate. This means the risk of hospitalization and/or ED visits may increase drastically when an older adult’s heart rate exceeds 83.24. Thus, our study highlights the clinical importance of heart rate related to hospitalization and/or ED visits among older adults receiving home healthcare services. Similarly, previous studies have also examined the clinical importance of heart rate in the general population (Bui et al., 2013; Zhang et al., 2016). For example, a meta-analysis found that high heart rate is associated with cardiovascular and all-cause mortality in the general population (Zhang et al., 2016). As such, older adults need to be monitored for any changes in their vital signs, especially the heart rate, from their baseline. Clinicians need to understand the importance of continuous assessment and monitoring of vital signs during their home visits, especially regarding the identified critical point of heart rate, to reduce the risk of future hospitalization and/or ED visits. In addition, they need to monitor any subtle changes in their heart rate that may indicate important clinical warning signs and warrant additional evaluation (Chester & Rudolph, 2011).

There are several limitations to consider. First, our study considered systolic blood pressure, diastolic blood pressure, and heart rate for the current study. Since the GAM model depicts only the nonlinear relationship, we did not consider other vital signs, such as temperature, that showed a linear relationship with hospitalization and/or ED visits among older adults receiving home healthcare services. Other vital signs also serve as important indicators of their clinical status, and future research should consider using other methodologies to identify a critical point for these vital signs. Second, this study relied on baseline vital sign measurements taken at the time of admission to home healthcare, as these are consistently recorded for nearly all patients. Given that vital signs can be influenced by various factors, future research should investigate the temporal patterns of these vital signs and their longitudinal association with the risk of hospitalization and/or ED visits. In addition, future studies should also consider examining the vital sign readings from acute care settings or incorporating remote patient monitoring values from telehealth to gather a more comprehensive understanding. Third, older adults are a heterogeneous group and are further classified into young–old (65–74 years), old–old (75–84 years), and oldest–old (85 years and over). Thus, future research may consider examining the similarities and differences in their relationship between vital signs and hospitalization and/or ED visits and the critical point for each vital sign across the three groups.

## Conclusions

This study employed GAM to investigate the nonlinear correlations between vital signs and the risk of hospitalization and/or ED visits in older adults receiving home healthcare. Critical inflection points were identified for each vital sign, with heart rate showing the most marked nonlinearity, which highlights the need to consider the nonlinearity in future studies. Our findings reveal that the risk of hospitalization and/or ED visits sharply increases when an older adult’s heart rate surpasses 83.24 bpm. Therefore, clinicians must be cognizant of these critical thresholds for each vital sign and diligently monitor any deviations from baseline to preempt adverse outcomes.
